# Disease-specific data processing: An intelligent digital platform for diabetes based on model prediction and data analysis utilizing big data technology

**DOI:** 10.3389/fpubh.2022.1053269

**Published:** 2022-12-12

**Authors:** Xiangyong Kong, Ruiyang Peng, Huajie Dai, Yichi Li, Yanzhuan Lu, Xiaohan Sun, Bozhong Zheng, Yuze Wang, Zhiyun Zhao, Shaolin Liang, Min Xu

**Affiliations:** ^1^School of Health Science and Engineering, University of Shanghai for Science and Technology, Shanghai, China; ^2^Department of Endocrine and Metabolic Diseases, Ruijin Hospital, Shanghai Institute of Endocrine and Metabolic Diseases, Shanghai Jiao Tong University School of Medicine, Shanghai, China; ^3^School of Public Health, Li Ka Shing Faculty of Medicine, The University of Hong Kong, Hong Kong, Hong Kong SAR, China; ^4^School of Food Science, Shihezi University, Shihezi, China; ^5^STI-Zhilian Research Institute for Innovation and Digital Health, Beijing, China; ^6^Institute for Six-sector Economy, Fudan University, Shanghai, China

**Keywords:** medical data processing, model prediction, diabetes, platform construction, digital therapeutics

## Abstract

**Background:**

Artificial intelligence technology has become a mainstream trend in the development of medical informatization. Because of the complex structure and a large amount of medical data generated in the current medical informatization process, big data technology to assist doctors in scientific research and analysis and obtain high-value information has become indispensable for medical and scientific research.

**Methods:**

This study aims to discuss the architecture of diabetes intelligent digital platform by analyzing existing data mining methods and platform building experience in the medical field, using a large data platform building technology utilizing the Hadoop system, model prediction, and data processing analysis methods based on the principles of statistics and machine learning. We propose three major building mechanisms, namely the medical data integration and governance mechanism (DCM), data sharing and privacy protection mechanism (DPM), and medical application and medical research mechanism (MCM), to break down the barriers between traditional medical research and digital medical research. Additionally, we built an efficient and convenient intelligent diabetes model prediction and data analysis platform for clinical research.

**Results:**

Research results from this platform are currently applied to medical research at Shanghai T Hospital. In terms of performance, the platform runs smoothly and is capable of handling massive amounts of medical data in real-time. In terms of functions, data acquisition, cleaning, and mining are all integrated into the system. Through a simple and intuitive interface operation, medical and scientific research data can be processed and analyzed conveniently and quickly.

**Conclusions:**

The platform can serve as an auxiliary tool for medical personnel and promote the development of medical informatization and scientific research. Also, the platform may provide the opportunity to deliver evidence-based digital therapeutics and support digital healthcare services for future medicine.

## Introduction

In spite of medical advancements in the 21st century, a major health crisis remains unresolved. The prevalence of diabetes mellitus has increased globally over the past decades, despite improvements in sanitation, antibiotics, vaccines, and other medical interventions (1). Diabetes mellitus is a major public health problem worldwide (1).

The World Health Organization released the world's first health report on diabetes on the 7th World Health Day, noting that the disease has affected human health and development over the past few decades (2). According to survey statistics, the incidence of diabetes is the highest in developing countries, especially in Asia, where India and China have the most cases. These large populations and high prevalence rates have imposed heavy economic and social burdens on developing countries. Statistics show that, as of 2021, one person in eight has diabetes in China, making diabetes one of China's crucial public health issues.

In order to reduce the burden of diabetes disease on society and individuals, it is recommended that individuals over the age of 45 with risk factors for diabetes do preventive and pre-diabetic screenings, as well as regular monitoring for those who already have diabetes, which has become a key point to alleviate the problem. At the same time, in order to prevent or delay the development of type 2 diabetes mellitus, lifestyle interventions as well as diabetes self-management education and support are also recommended (3). Given the current pandemic setting and unprecedented situation worldwide, digital technology-assisted interventions are recommended for the prevention of diabetes mellitus, which has become a current research hotspot.

Different forms of digital therapeutics such as online platforms, virtual reality trainings, and applications applying technological solutions to enhance healthcare are being tested the feasibility (4). The rapid growth of information, biological, and communication technologies makes this an opportune time to develop digital tools that deliver precision interventions for health behavior change to address the diabetes mellitus crisis. In recent years, systems related to clinical medical data, such as hospital information systems (HIS), clinical information systems (CIS), laboratory information systems (LIS), and electronic medical record (EMR), have accumulated large amounts of medical data. With the continuous growth of medical data, the value of the data has gradually become apparent.

However, this also brings some challenges to digital medicine. A large amount of patient data information poured into the medical database, making the amount of medical data increase exponentially. These large-scale data are accompanied by a variety of complex data forms at the same time, which increases the difficulty of studying the value of these data in terms of data quality and data structure. In the system construction of traditional hospital information platform, it often includes four layers: hardware network infrastructure layer, datacenter data layer, business service layer, and data exchange layer. This traditional system construction tends to be more inclined on the service side, so the analysis and processing process of massive stored data is lacking at the doctor-patient service business level supported by big data exchange and storage technology, and it is difficult to tap the huge value behind medical data.

Based on the above discussion, this project focuses on the research of big data processing and analysis platform based on data mining. By focusing on the latest research focus of current big data technology, this research creatively proposes three major platform building mechanisms, namely DPM, DCM, and MCM. At the same time, the whole platform is structured in combination with big data platform building technology. Finally, we developed a processing and analysis platform based on real-world intelligent clinical data of diabetic patients. This platform is more suitable for complex clinical data analysis. As an adjunct to diabetes early warning, prevention, early detection and treatment, which have significant clinical significance and contribute to the improvement of people's health. In the following part of this paper, we will introduce and expound on the research foundation and application of the platform, which can serve as a tool for early prevention and diagnosis of diabetes in China, as well as a practical reference for researchers.

## Related work in the field of big data

### Existing related work at home and abroad

With the rapid development of information technology, the value of medical data has attracted the attention of many scholars worldwide. As early as 1969, Greenes et al. (5) designed and developed a clinical data management system in the United States to improve medical records' manual entry mode that has been used for centuries. This system has a highly flexible environment interface and can process variable-length text string data and store the organized structure of files in a tree structure. This medical information system that can standardize clinical data, ensure the quality of the data, and allow retrieval of the data has extensively influenced the development of medical informatization. Since then, Ng et al. (6) designed and developed the “PARAMO” system as a predictive modeling platform for analyzing electronic health data. PARAMO supports the generation and reuse of clinical data analysis patterns for different modeling purposes. Additionally, to process parallel tasks efficiently, PARAMO established a large-scale data analysis model based on MapReduce, which can analyze large amounts of medical data and process them in a reasonable time. Moreover, Ng et al. integrated medical terminology ontologies (such as ICD and UMLS) into the PARAMO system. When testing the patient sample information in the dataset, it was found that the execution speed of concurrent tasks was significantly improved in this extensive dataset system.

Over time, clinical data analysis has increasingly been oriented toward using systems such as Electronic Health Records (EHRs) and Clinical Information System (CIS) to develop predictive models for different patient groups. Predicting disease risk and progression plays an essential role in clinical decision support; however, developing a computational model for clinical prediction requires complex schema construction. Zolfaghar et al. (7) conducted a study on the risk of 30-day readmission in patients with congestive heart failure using a big data technology scheme by extracting data from the National Inpatient Data Set (NIS) and the Multicenter Health System (MHS). A hierarchical logistic regression model and a random forest algorithm model were constructed to predict the probability of patient readmission. The researchers tested data from more than three million medical records in multiple scenes at different stages. The results show that the effectiveness of the comprehensive data-based open-source prediction modeling framework significantly improves the performance of predictive modeling. In addition, Deligiannis et al. (8) proposed a prototype data-parallel algorithm to reduce the risk of sudden cardiac death among young athletes and promote the successful diagnosis of mild hypertrophic cardiomyopathy (HCM). A rule-based machine learning approach was used to diagnose large datasets using iterative MapReduce. A successful diagnosis of HCM is highly challenging because of the many latent variables presented in HCM. Deligiannis et al. believed that the diagnosis rate can be improved through data-driven analysis. At the same time, the experimental results showed that the overall runtime of predictive analytics reduced from 9 h to just a few min when accessing a dataset containing 10,000 accurate medical records. This is a significant improvement over previous analyses, potentially enabling the technology to be used for the systematic diagnosis of early disease in the future.

In addition, the use of big data technology to analyze clinical disease data has a significant impact on the medical community. By collecting data from patients inside and outside the hospital, analyzing and determining the causal relationship between different disease symptoms, and determining disease risk prediction models, medical optimization and patient health management will be greatly enhanced.

### How to make good use of data mining technology in the field of diabetes

Diabetes, a global problem, has become one of the three biggest threats to human health. Patients with diabetes who do not receive adequate treatment will develop cardiopulmonary diseases, liver complications, nerve damage, etc., which can seriously affect their health (9). In this situation, early diagnosis and prevention of diabetes are critical.

Thakkar et al. (10) applied data mining and fuzzy-logic techniques to a diabetes diagnosis. They combined machine learning and statistical methods to improve the accuracy of their algorithm through feature selection. Finally, 99.7% prediction accuracy was achieved using a stochastic forest classifier. A fuzzy logic system was introduced to deal with the uncertainty in medical diagnosis data, and fuzzy data analysis was performed using highly approximate linguistic concepts. Thakkar et al. found that high accuracy and low complexity contribute to 96% accuracy in the case of many fuzzy logic methods, which is essential for early diagnosis and prevention of diabetes. In terms of platform and system, Sivaparthipan et al. (11) proposed a healthcare information system model based on the map function and the reduced function in the Hadoop architecture, which utilizes big data technology to analyze and assess diabetes. They migrate data to different parts of the system and process it through different information blocks and centers to predict different types of diabetes and provide effective treatments. The platform evaluates the system through the precision of the statistical evaluation model and the performance index, such as the F-measure. The ANN algorithm of the artificial neural network in the system achieved a precision of 0.988, and the highest index evaluation value based on F-measure achieved a precision of 0.96. This further proves the effectiveness of the system, which is better than that of the existing methods.

Patients can benefit from this clinical disease risk assessment system in terms of early diagnosis, treatment, risk assessment, and disease prediction. It is essential for patients with chronic diseases such as diabetes to improve medical diagnosis and treatment by using big data technology based on artificial intelligence, strengthening the education of patients, and providing the corresponding health monitoring function (12, 13). However, in medical informatics based on clinical information data, it is challenging to construct a disease risk prediction and assessment model because the medical data are extensive and the data dimension is complex (14, 15). This paper focuses on building and demonstrating how to use big data technology to implement and extend the big data processing and analysis platform based on chronic diabetes diseases to solve these problems.

## Architecture of diabetes clinical big data processing and analysis platform

### Overall system architecture

**Figure 1:**
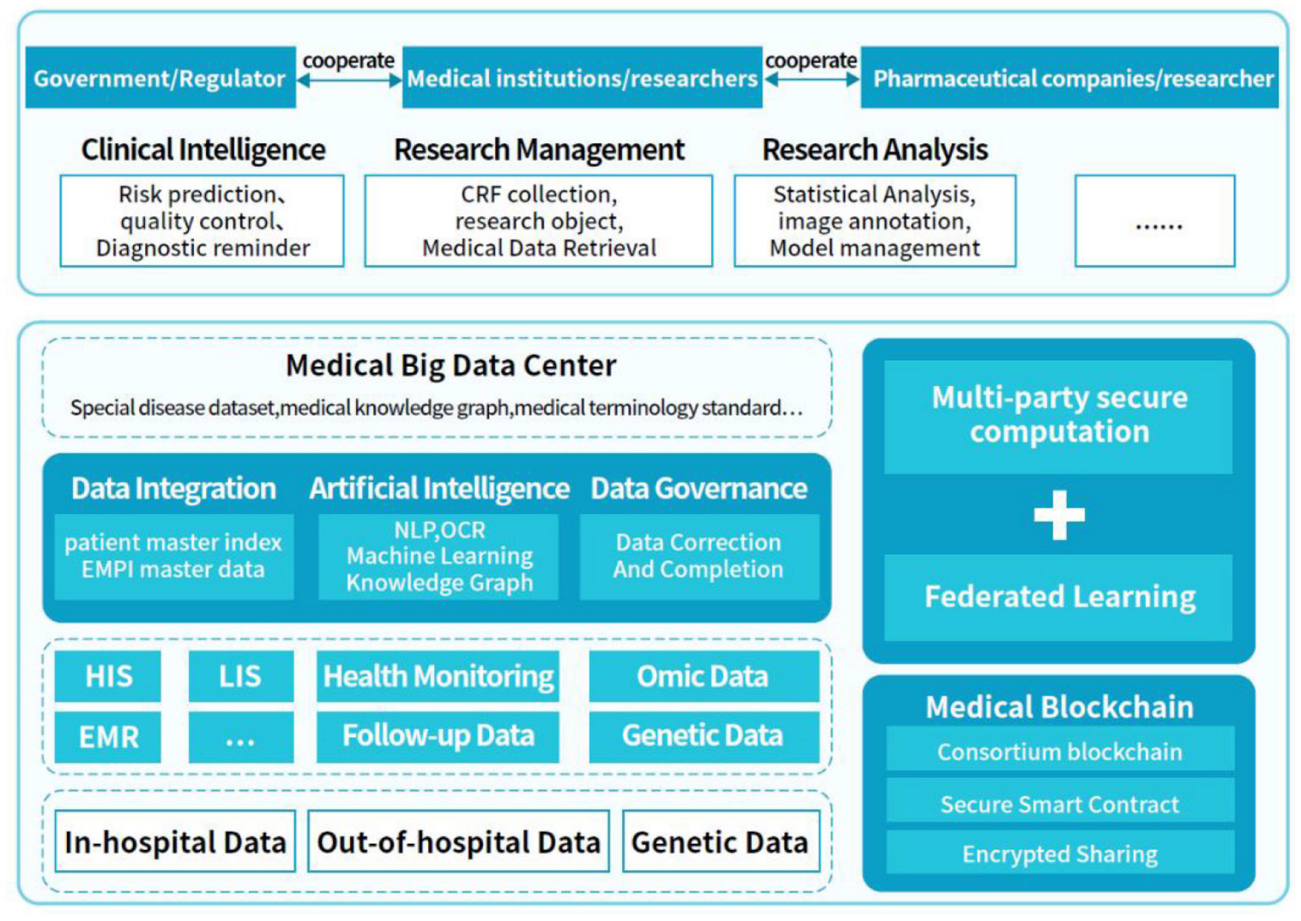
RDDP platform architecture diagram.

Because the traditional medical diagnosis of diseases often has a high demand for doctors' diagnostic experience and subjectivity, it is easy to produce misdiagnosis and missed diagnosis. Moreover, traditional medicine needs large human and material resources, and the distribution of medical resources in different regions is often unbalanced. At the same time, it is difficult to promote medical research by virtue of digital development. Based on these problems, in order to break the barriers between traditional medical research and digital medical research, and promote the development of medicine. This section proposes a treatment and analysis platform based on the clinical Real-world Data of Patients with Diabetes (RDDP). The architecture of the platform is mainly composed of three modules, as shown in Figure 1: medical data integration and governance (DCM), data sharing and privacy protection (DPM), and medical application and medical research (MCM). The data sources in the DCM mechanism mainly come from the data inside and outside the hospital of each cooperative unit. These include in-hospital HIS, LIS, EMR data, out-of-hospital health monitoring data, and follow-up data based on intelligent medical hardware and the medical Internet of Things. It also included genomic and genetic sample data. Data integration, data governance, data mining, and other processes are used to extract value from data, finally, a medical data center is built to provide support for the entire system through data processing.

The DPM mechanism mainly realizes data sharing and privacy protection. In this section, data security is traced, encrypted, and shared by blockchain technology and combined with a multiparty security computing model and federated learning algorithm to realize multiparty joint queries, statistics, and joint modeling, providing a security guarantee for the entire system. The MCM mechanism is a system application scene terminal based on the DCM and DPM mechanisms, including parts of intelligent clinical medical application and medical research application, in which the government, medical institutions, and pharmaceutical companies collaborate. The platform can be used in intelligent clinical supervision, medical data statistical analysis, and scientific research management, which offers a means for bridging the gap between traditional medical treatment, digital medical treatment, and medical research to promote the development of medical informatization and medical research.

### Data storage and management

**Figure 2:**
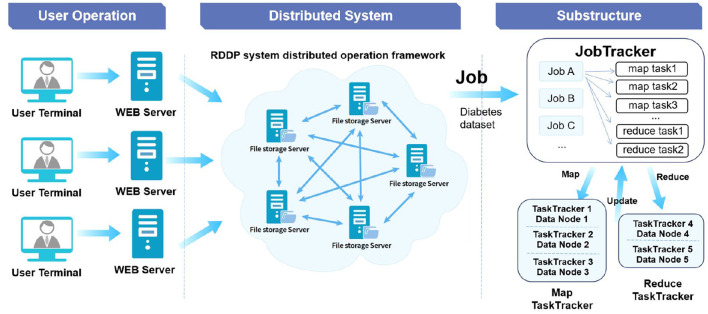
RDDP system distributed storage architecture.

The data stored in medical information systems often have the characteristics of enormous data volumes, strong data dispersion, multiple data types, incompatible versions, complex data structures, and high data privacy security. In order to be able to adapt to these characteristics of medical data in the process of data storage and processing, this system adopts a distributed data storage scheme, as shown in Figure 2. Through the decentralized storage method, diabetes data information in the system is stored in different unit blocks to enhance data security and flexibility.

In addition, the system implements a distributed storage solution based on Hadoop Distributed File System (HDFS) and Hadoop Database (HBase) technologies of the Hadoop system. We choose to use different storage schemes for different use scenarios. For data sets that often need to be modified and written, such as data in each stage of data processing, we use HBase technology for data storage management. At the same time, HBase technology is used to store some structured and semi-structured data. When mass data query and data appending operations are required, such as bulk medical original data sets, HDFS technology is used to scan and append the data. The distributed file system based on HDFS stores the files in Namenode and Datanode and achieves file partition and sending and reading through the HDFS Client to ensure the entire system's stability. HDFS also provides HBase with highly reliable underlying storage support to meet the underlying storage function of big data on HBase.

This distributed file processing system has the characteristics of high throughput and high access volume. It can be strongly adapted to applying clinical data such as diabetes disease data sets. Meanwhile, HBase adopts the column family method in data storage, which combines the business advantages of OLTP and OLAP, supports a large number of concurrent users to process data simultaneously, and ensures the efficiency and consistency of data analysis (16).

### Data distributed processing

To ensure the system's stability in processing a large amount of medical information data, our system adopts a distributed data processing scheme based on MapReduce while using distributed data storage to realize the intelligent mining of diabetes data. JobClient generates the task run file in the MapReduce scenario, which JobTrack splits and sends to JobinProgress of the TaskTracker program and TaskScheduler. In addition, Jobin Progress further decomposes the job into Map and Reduce computation and places them in the TaskTracker server. Map and Reduce computing are two cores of data distributed processing. Map is to decompose a complex task into several easy to process tasks for execution. Reduce is a process of summarizing the results of the Map phase. For example, when we are building a classification prediction model for medical diseases, we can divide the final classification problem into several different submodels for construction, and finally summarize the results of different stages to obtain the final prediction value. Through this technical method of data distributed processing, it provides computing power for diabetes data mining.

### Intelligent data mining

The data intelligent mining part aims to realize information mining of diabetes clinical data based on artificial intelligence technology. For data governance of complex clinical data information, the corresponding data mining algorithm models are selected according to the different research purposes of researchers, such as the XGBoost algorithm (17) based on decision tree optimization, the LightGBM algorithm (18), and the K-NearestNeighbor (KNN) algorithm (19) for missing value processing of medical data. At the same time, in future research, we will conduct further model establishment and algorithm research on other machine learning methods such as Support Vector Machine (SVM) (19), random forest, logistic regression (18), and deep learning network models (20). It then realizes important feature screening, disease prediction, risk assessment, etc. Of clinical diabetes and other related diseases, and extracts decision–making indicators and programs related to diabetes diagnosis and treatment. The RDDP system is used to assist in diagnosing and treating diabetes in clinical medicine and provide the necessary data and statistical analysis functions for researchers.

### System interaction and visualization

To improve the intelligence and interactivity of the RDDP system, the data visualization part is added after the intelligent data mining process (21). The data information can be displayed more intuitively through statistical analysis and display methods, such as statistical maps, heat maps, and word cloud drawing. With this friendly and straightforward method, users can interact more effectively with the system, and clinicians and researchers can explore the value of the data more effectively, resulting in improved patient outcomes.

### Data integration

To better deal with the distribution and heterogeneity of clinical medical data, methods such as federation, middleware-based models, and data warehouses are typically used to construct a data integration system. In the data integration scheme of this project, we focus on the four principles of data consistency, security, stability and scalability. At the same time, we integrate data according to two categories of data integration systems, namely, materialized integration system and virtual integration system. We integrate the data in the medical information system, including HIS, LIS and CIS systems, virtually, and incorporate other medical data into the data set of this system through the materialization integration method. This system's data storage and processing are composed of HDFS and HBase systems based on the Hadoop framework. This data integration system constructed using the data warehouse method can effectively process and mine data of different structure types. Through technologies such as Flume and Sqoop, the related data of different data sources can be integrated into the HDFS cluster, which can meet the performance requirements of the RDDP system for diabetes extensive data integration and then provide data support for the big clinical data scientific research management platform.

### Data security and privacy

The security and privacy of medical data are integral parts of the current medical informatization construction, including the security of medical information in data integration and transmission and the privacy protection of patients' personal medical information. Based on this, in addition to ensuring the security of the mapper in the Hadoop distributed framework, the system adds the feature of ensuring the traceability and non-tampering of medical data by using blockchain technology, enabling privacy and security processing. At the same time, combined with privacy computing technology, on the basis of building a distributed storage database, we can achieve distributed joint and statistical analysis. Use multi-party security computing technology to achieve joint analysis and sharing.

For patient sensitive information, we store it in the cloud through homomorphic encryption, and perform operations based on ciphertext, such as query, retrieval, statistics, etc. The result of the operation is still in the form of ciphertext, and the result is returned through the cloud. The whole processing process is in the process of encryption, which effectively protects patient privacy. Meanwhile, the system is developed through the user rights management module in terms of construction, and the security of user information is guaranteed through authentication, verification, access control, and other methods.

## Implementation of diabetes clinical big data processing and analysis system

**Figure 3:**
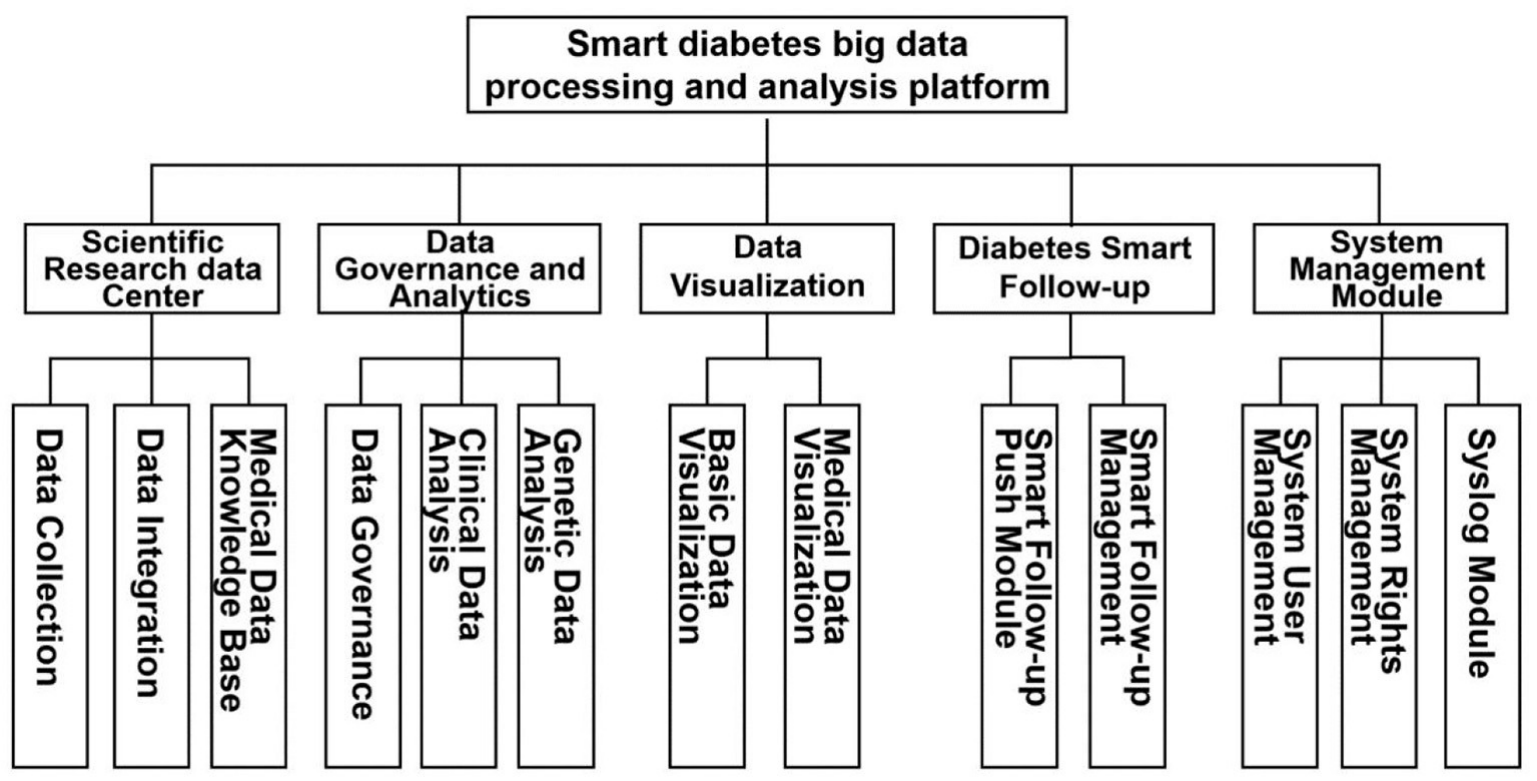
Functional block diagram of RDDP system.

The RDDP platform comprises five modules: the scientific research data center module, the data governance, and analysis module, the data visualization module, the intelligent follow-up module, and the system management module. The scientific research data center module mainly provides medical dataset support for the platform, ensures the average upload and download of platform data, and additionally provides medical data dictionaries for platform users. The data governance and analysis module mainly cleans and analyzes the data to be analyzed by the user according to the data characteristics and user needs and then visually presented to the user after statistical analysis. At the same time, through the design and implementation of the diabetes intelligent follow-up module, the system strengthens the communication between doctors and diabetic patients so that doctors can gain a deeper understanding of patients' current health status and provide corresponding diagnoses and treatment Suggestions. Specific functional modules are shown in the following Figure 3.

### Clinical research data center

**Figure 4:**
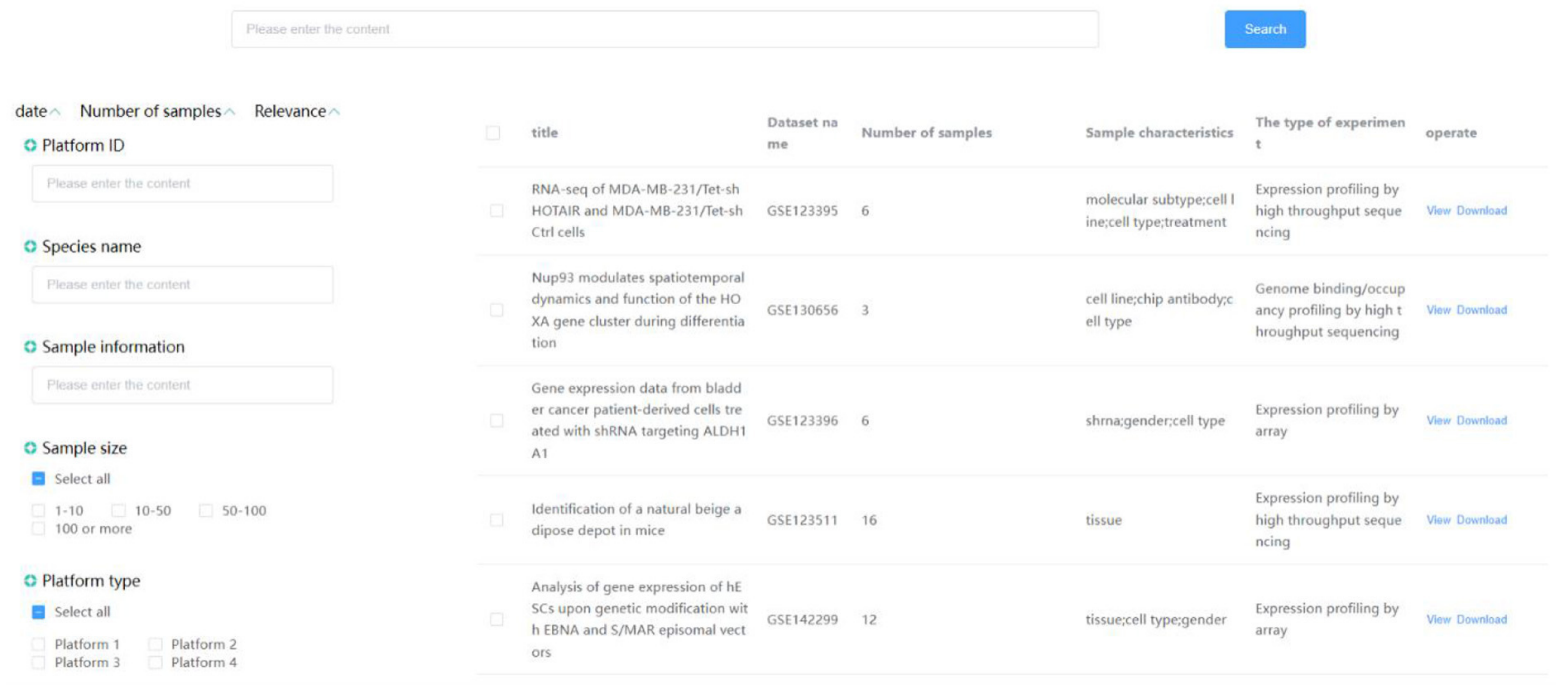
Clinical data center interface.

Integrate data from diabetes, clinical outpatients, HIS, LIS, EMR and other systems, including structured/unstructured/semi-structured data such as medical images, electronic medical records, text data, into the clinical research data center. By establishing a standardized normative system, the data are imported in a structured and standardized manner. The Enterprise Master Patient Index (EMPI) is established using the cross-index algorithm to realize the fusion of multi-source data and information exchange. Finally, the generated metadata is authorized and deprived. The data is stored in the intelligent diabetes big data processing and analysis platform to enable the collection, integration, and support of multi-source data. The system interface is shown in Figure 4, which shows the medical data information integrated in the system.

**Figure 5:**
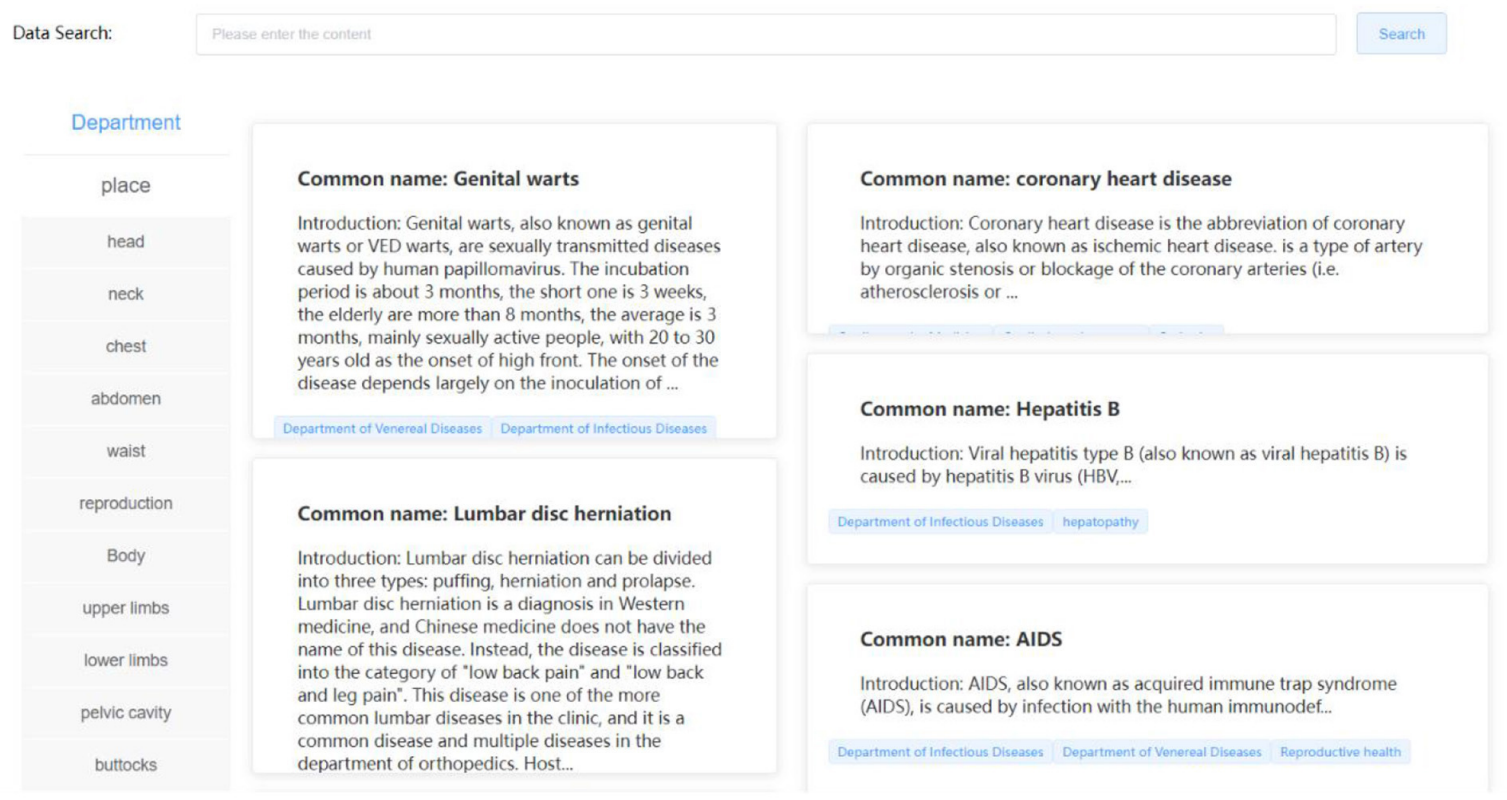
Medical knowledge base interface.

The medical knowledge base is formed by processing the actual diabetes knowledge, clinical medical disease, drug information, and health care mutual-related data through data cleaning work, such as data missing value filling, data replacement, and data screening to form high-quality, multidimensional structured data to meet the needs of scientific research retrieval, medical knowledge popularization, data understanding, and other related functions. At the same time, in this section, we use web crawler technology to automatically crawl medical knowledge information according to specific fields without affecting the intellectual property rights of related websites and organize it into medical information with high utilization value and popular science functions. As shown in Figure 5, the rich medical data knowledge base is displayed.

### Data governance and analysis

**Figure 6:**
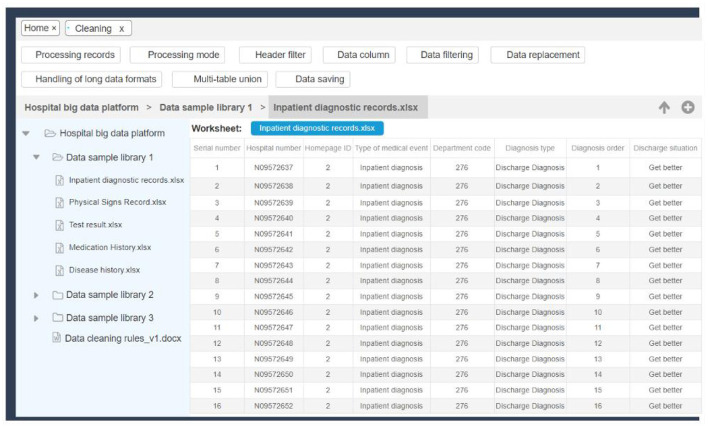
Data governance interface.

In the data governance and analysis section, the system implements data cleaning operations, including data screening, data replacement, deviation correction, and completion, by constructing data processing methods based on natural language processing, statistical principles, and machine learning. The establishment of these methods provides governance tools for medical data from complex to unified. The specific interface is shown in Figure 6, which shows a basic sample data and some data governance functions.

Simultaneously, by establishing statistical analysis models based on artificial intelligence algorithms in this system, including matrix correlation analysis, univariate survival analysis, and bivariate distribution analysis, the data of clinical diseases, including diabetes and other related diseases, were analyzed. Specific patterns are mined from the data for analysis, using box plots, radar charts, scatterplots, pie charts, and other visual graphics to present the hidden information and laws in the data, creating interactive views.

**Figure 7:**
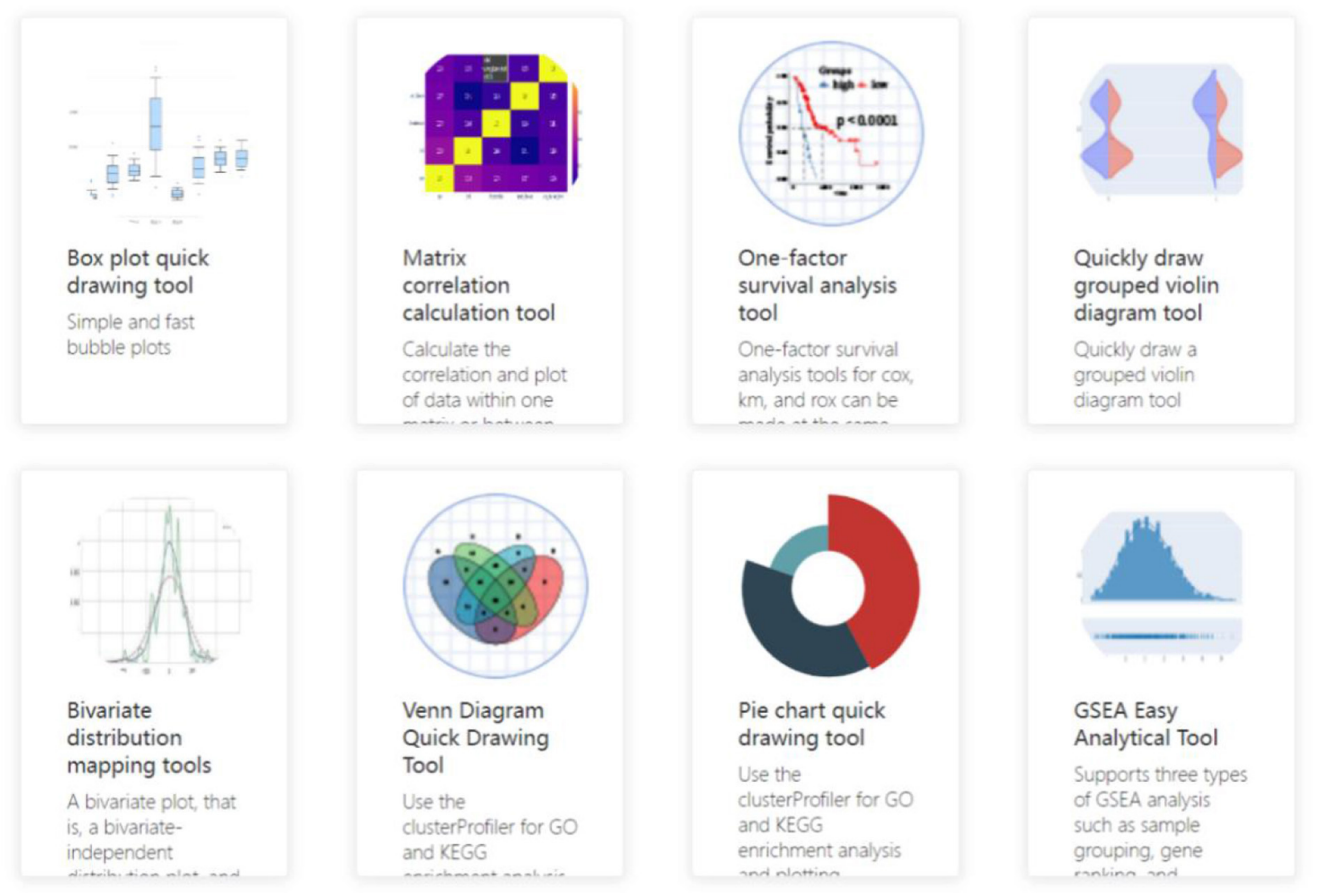
Data analysis tool interface.

**Figure 8:**
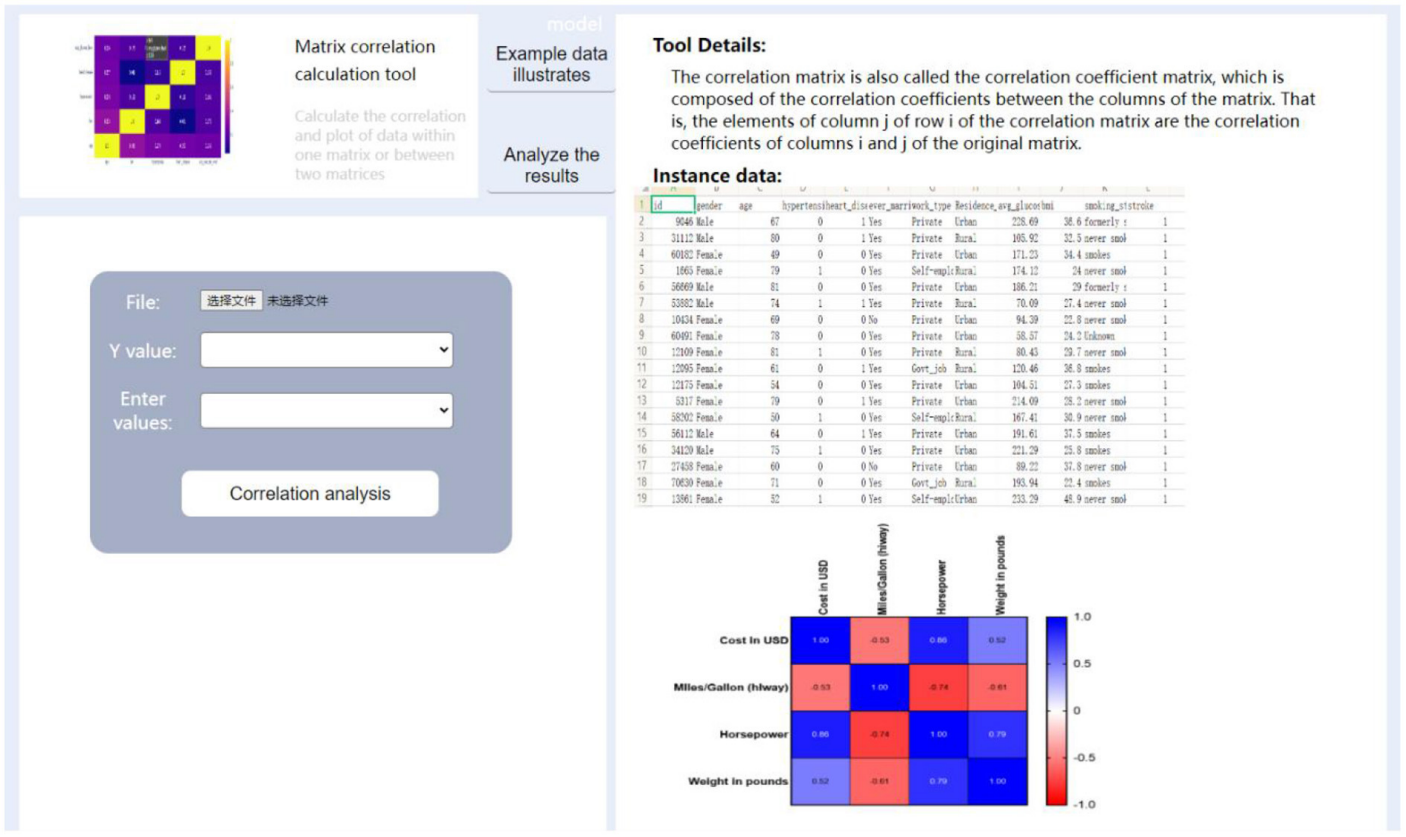
Data analysis tool details interface.

In the follow-up, we will establish models including batch survival analysis, multi-data set analysis, and limma rapid differential analysis through further experiments to ensure the integrity of the function of this plate. The entire process is then completed: the design of the management, statistics, analysis, and visualization of user data. The actual R&D interface for this part is shown in Figures 7, 8, which show some commonly used medical analysis tools we integrate into the system and their specific operation pages.

### Data visualization

**Figure 9:**
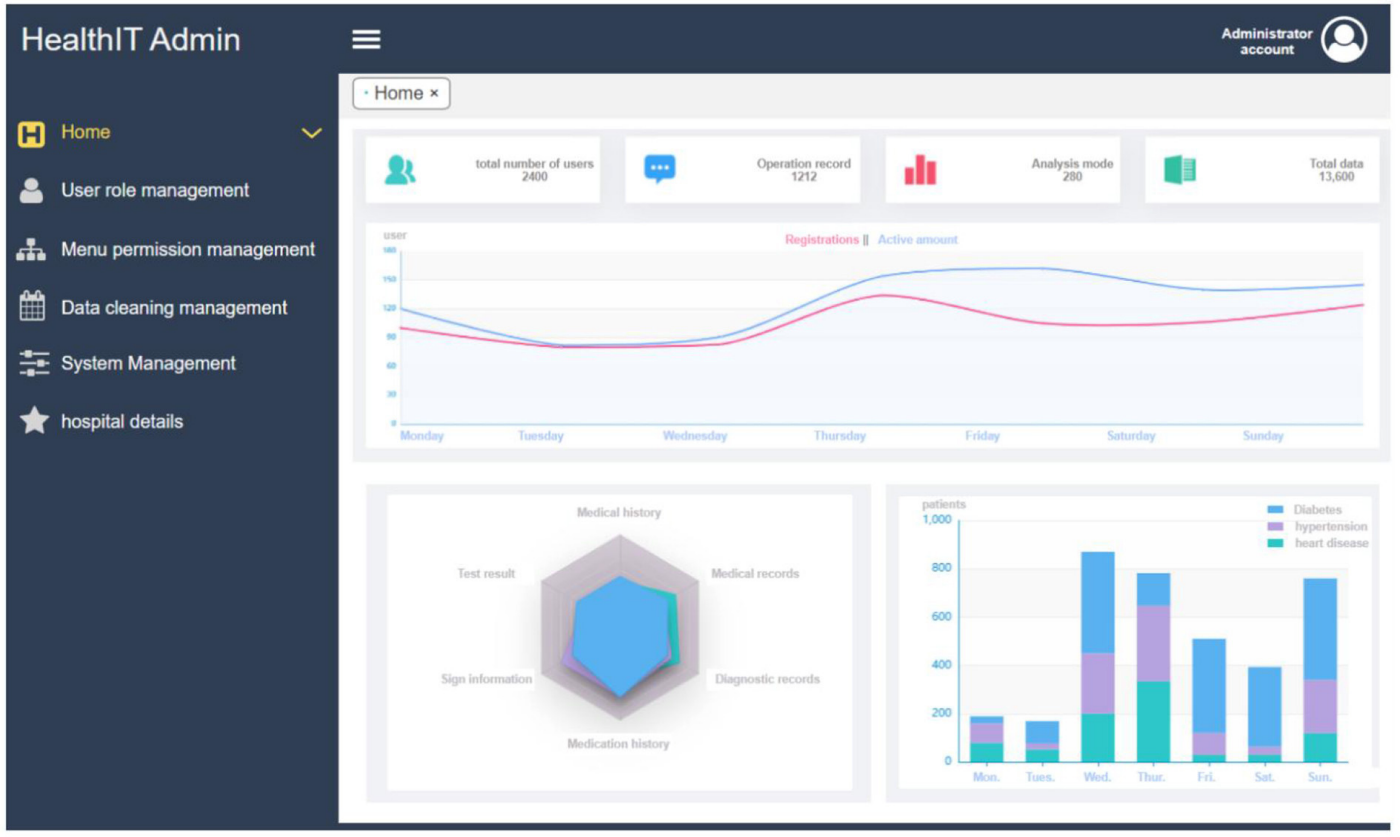
Data visualization interface.

The data visualization part provides the visualization interface of the two modules, including basic information visualization and disease data visualization. In the data visualization module, we built 2D and 3D visualization graphics images using a Python-based drawing library, including Matplotlib and Seaborn. The medical data can be displayed in a more intuitive way in front of the user, including data histograms, box diagrams, violin diagrams, scatterplots, heat diagrams, and other display methods. In this module, a visual representation of the basic information of the platform provides information on the number of users, follow-up records, analysis modes, and clinical data of the platform. In addition, the registration and activity of the platform were displayed in the form of real-time statistics. Disease data visualization mainly displays disease history, sign information, diagnosis records, test results, physical examination records, and other relevant contents. The specific interface is shown in Figure 9, which shows the basic user data information, the statistical classification information of diseases and the daily registered users/active amount of the system.

### Intelligent follow-up of diabetes

**Figure 10:**
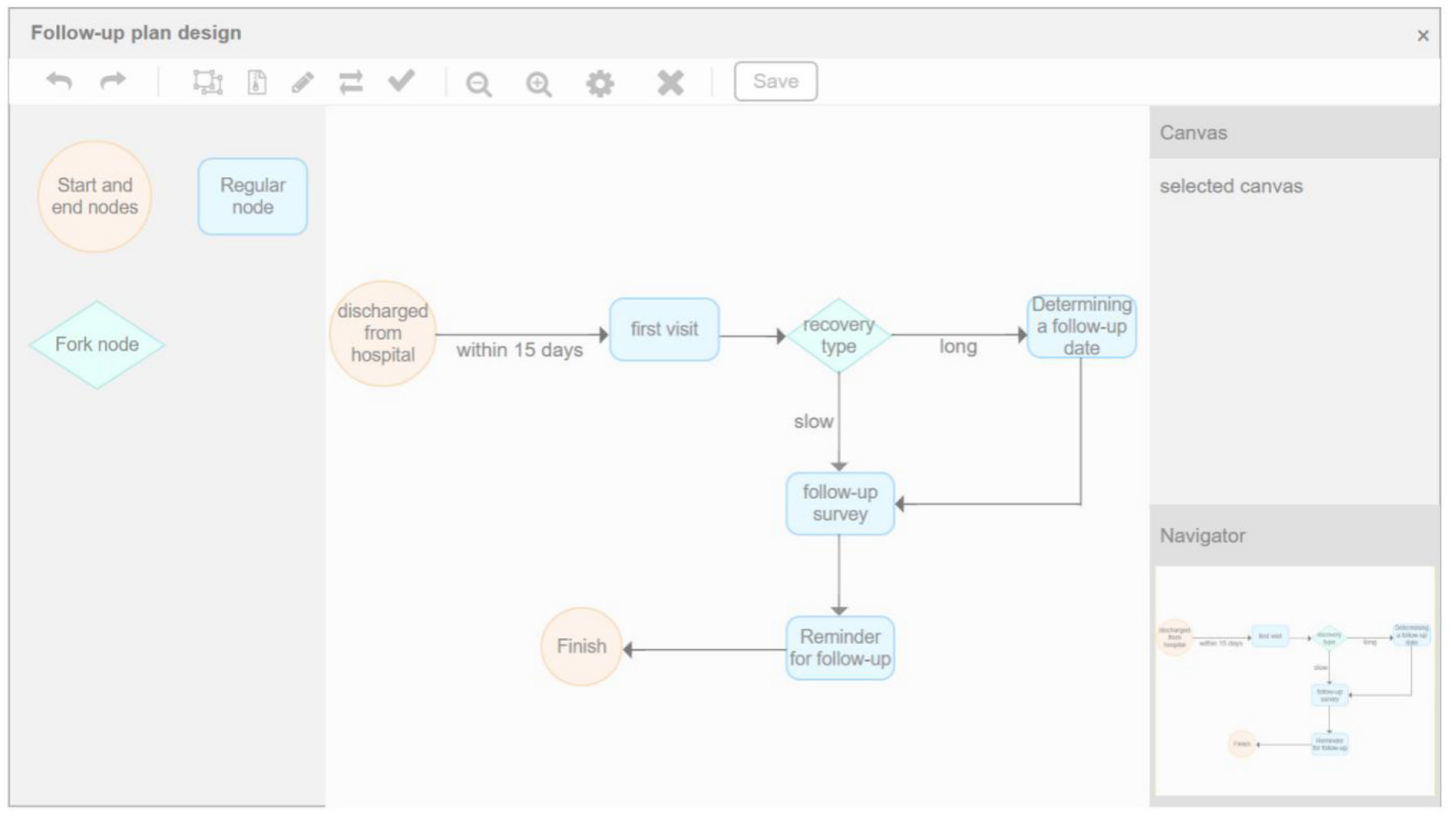
Diabetes smart follow-up.

**Figure 11:**
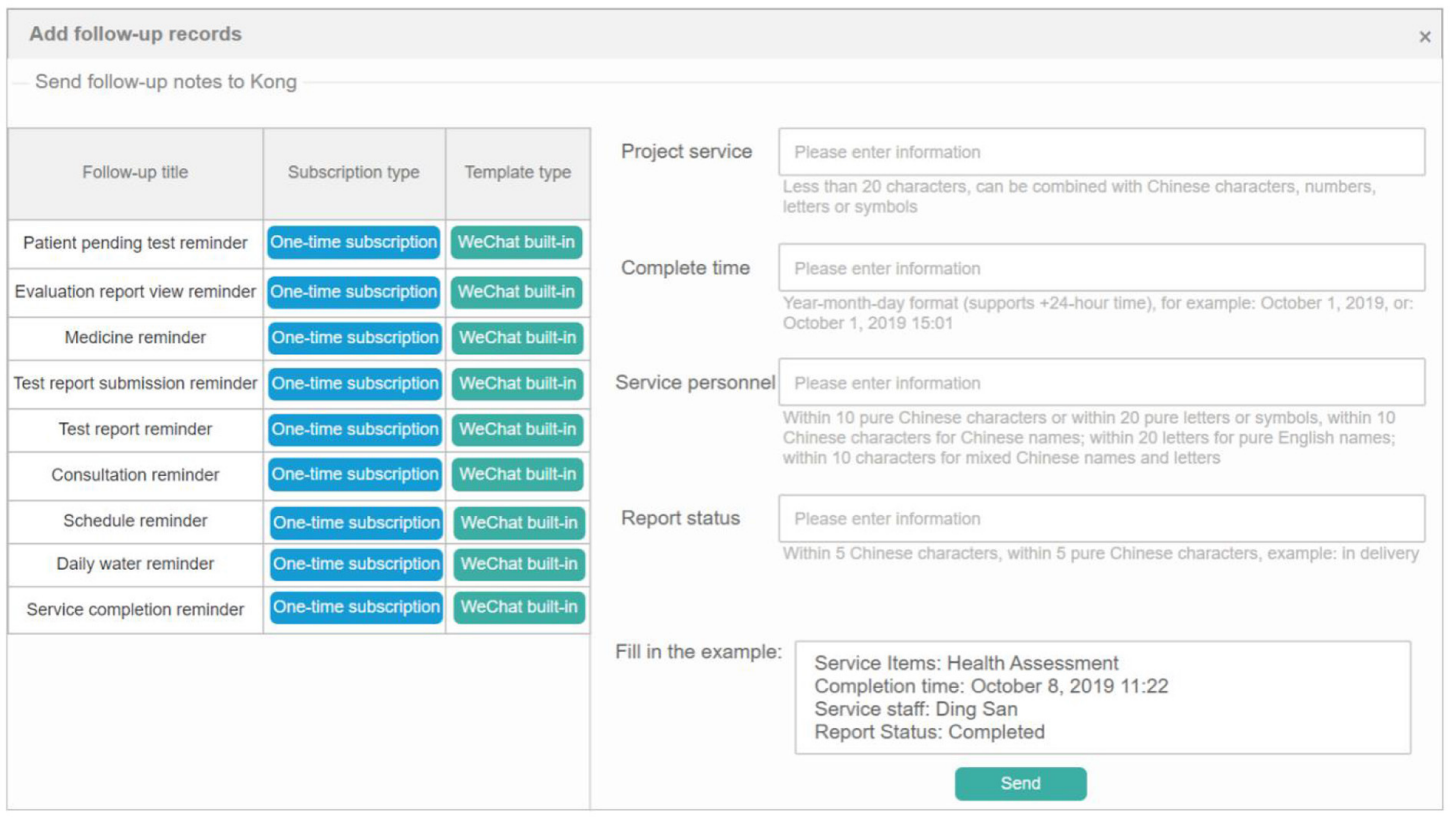
Follow-up record management.

Intelligent follow-up module provides a follow-up template library covering 22 common departments and their key diseases, combined with custom follow-up plan templates, to meet the doctors' regular follow-up function for diabetic patients. Meanwhile, through the online follow-up function, the customer's follow-up data was managed. Intelligent matching of follow-up plans and management plans provides customers with a variety of online communication modes, formulates evaluation guidance, and conducts statistics and analysis of user follow-up data through the system to comprehensively, conveniently, and intelligently serve the health needs of customers. As shown in Figures 10, 11 below, which shows the specific interface of the intelligent follow-up module for diabetes.

## Discussion

Applications of big data analytics technology is a good way to improve patient-centered care, detect disease outbreaks earlier, generate new insights into how diseases are spread, monitor medical and healthcare institution quality, and provide more effective treatment. Since this platform can be used to analyze complex data, provide early warning and prevention, detect diabetes diseases early and treat them, it holds value as a tool to transform health care data into actionable clinical interventions. It is still a worldwide public health emergency to deal with diabetes mellitus, and even though some clinical trials have proven the effectiveness of several different forms of digital therapeutics in preventing diabetes mellitus, there are few approaches to prevent Mellitus diabetes based on big data analytics (21).

The use of disease-related data analysis model can assist in identifying clinical interventions, reducing adverse events, and improving precision medicine and patient management. In light of the risk factors found in the research, performing regular health checks, risk assessments, and individualized interventions enduringly, it may be possible to decrease the incidence of diabetes mellitus or delay the progress of diseases with data-based guidance (22).

## Conclusion

The intelligent diabetes big data processing and analysis system developed in this project is based on clinical real-world data. Artificial intelligence technology is used to mine massive amounts of medical data, aiming at the characteristics of high data complexity, large data volume, and high data sensitivity requirements in clinical data. In the system framework, a distributed data storage and management scheme was established, and diabetes data located in different data sources was integrated into the unit location data storage to ensure the timeliness and stability of the system during operation. The design and implementation of this big data processing and analysis system based on artificial intelligence, the Internet of Things, and cloud computing can help clinicians, researchers, government agencies, etc. to effectively predict diabetes and other related diseases based on clinical data. Meanwhile, it can help in risk control and management of the disease.

In order to better optimize the system performance of this project and meet the processing and analysis of daily medical data, we will further evaluate the performance of this project in the next work and constantly improve the system. At the same time, the current focus of the project is on the research of diabetes related diseases. In future work, we will continue expanding other diabetes-related functions and diseases. We will iterate various disease systems and integrate some disease data mining functions to realize the informatization of common disease data according to different actual medical needs. Furthermore, we will use artificial intelligence computer technology and the current information technology to empower clinical medical care and scientific research, thereby supporting the rapid development of the country's medical industry.

## Data availability statement

The original contributions presented in the study are included in the article/supplementary material, further inquiries can be directed to the corresponding author/s.

## Author contributions

XK: supervision and validation. RP: project administration, conceptualization, formal analysis, investigation, data curation, methodology, writing–original draft, and visualization. HD: conceptualization. YLi: methodology, conceptualization, and investigation. YLu: software and methodology. XS: investigation. BZ and YW: resources. ZZ: methodology. SL: writing—review and editing. MX: supervision and funding acquisition. All authors contributed to the article and approved the submitted version.
